# Effects of impact noise on the hearing of military personnel

**DOI:** 10.1590/S1808-86942011000600011

**Published:** 2015-10-19

**Authors:** Adriana Betes Heupa, Claudia Giglio de Oliveira Gonçalves, Herton Coifman

**Affiliations:** 1 Master's degree in Communication Disorders, Tuiuti University, Parana. Speech therapist of the Military Police Hospital, Parana state; 2 Doctoral degree in Collective Health, UNICAMP. Faculty speech therapist, master's degree and doctoral degree on Communication Disorders, Tuiuti University, Parana; 3 Doctoral degree in otorhinolaryngology, Sao Paulo University. Medical otorhinolaryngologist, adjunct professor, Parana Federal University

**Keywords:** firearms, hearing loss, military personnel, noise-induced, public health

## Abstract

**Abstract:**

Shooting is an activity that exposes military personnel to noise impact, which may cause irreversible effects on hearing.

**Objective:**

To evaluate impact noise on the hearing of military personnel that practice shooting.

**Study design:**

A case-control retrospective study.

**Methods:**

115 military personnel were enrolled; 65 had been exposed to impact noise and 50 were non-exposed. Firearm noise levels were evaluated, subjects answered a questionnaire and underwent threshold tonal audiometry and otoacoustic emissions testing.

**Results:**

The average noise level was 125dB(C). Most subjects (78%) believe that noise may cause hearing loss; nearly all (92.3%) used ear noise protectors while shooting, but most (32.3%) had never received guidance for using this equipment. There were significant differences between the two groups in relation to changes suggesting impact noise-induced hearing loss.

**Conclusion:**

The differences between groups show that noise-exposed military personnel are more likely to develop hearing loss. The goal of a hearing conservation program for this population should be to preserve hearing and educate these individuals about the importance of using hearing protection correctly.

## INTRODUCTION

Hearing abnormalities may result from exposure to elevated sound pressure levels, such as high intensity noise^1^. Acoustic trauma may occur following exposure to intense and short-lasting impact noise^2^,^3^.

Acoustic trauma consists of middle/inner ear injury caused by a single and short exposure to high intensity noise, such as an explosion. Its signs and symptoms are sudden hearing loss following exposure to noise, tinnitus, fullness of the ear, possible rupture of the tympanic membrane, and possible partial or complete destruction of the ossicular chain. Impact noise generally affects thresholds from 3,000 to 6,000 Hz; if exposure continues, other frequencies may be involved^2^,^4^.

One of the activities that expose individuals to high-intensity noise is handgun shooting, which is common in a military career. Depending on the activities in their career, military personnel may be exposed to continuous and/or intermittent noise (for instance, radio communicators, vehicle sirens, etc.) and to noise from firearms^5^.

There are studies on the intensity of firearm noise. A Greek study assessed the sound pressure level caused by military weapons and found sound peaks reaching 160.2 dBSPL^6^. In Brazil, the maximum sound pressure level of firearms used by shooting instructors in the Military Police of Montes Claros, MG, was found to be 108.9 dBSPL^7^.

The auditory effects of exposure to firearm noise have been investigated in several international studies^8^. A study in southern Brazil evaluated 101 military personnel that practiced handgun shooting and found that 20.79% of these subjects had sensorineural hearing loss, which is different from the incidence in civilians^9^. Another study in Sao Paulo described the symptoms in 72 firemen that were exposed to noise from 67 to 82 dBSPL during their work; the following complaints were found: irritability (20.8%), headache (14.5%), listening difficulty (14%), disordered sleep (13.5%), tinnitus (10.5%), and dizziness (1.5%)^10^.

Otoacoustic emissions (OAE) testing is an important tool for an early identification of noise induced hearing loss (NIHL). Outer hair cells in the inner ear are vulnerable to external effects such as noise; the first signs of cochlear abnormalities may be identified in OAE testing^11^. Several studies have assessed the efficacy of this test in military personnel exposed to noise.

A study in Israel evaluated OAE testing for the diagnosis of NIHL in military personnel, and revealed a clear association between auditory thresholds and OAE amplitude – worse auditory thresholds equate with lower amplitude. OAE testing is sensitive and objective and therefore provides valuable information for the diagnosis of NIHL and for supporting audiometry in the diagnosis and monitoring of cochlear function when exposed to noise^12^.

A Polish study of 92 military soldiers applied high-frequency audiometry and transient otoacoustic emissions (TOAE) before and after military duty. The conclusion was that TOAE testing was more sensitive than audiometry for the detection of NIHL^13^.

A Greek study consisted of verifying the effect of impact noise on distortion product otoacoustic emissions (DPOAE) in military personnel before and after exposure to firearm noise without ear protective equipment. The conclusion was that DPOAE may provide additional information about cochlear quality that may be used for monitoring purposes because it is a fast, objective, and easily done test^6^.

A study of military personnel in the Brazilian navy compared 60 individuals exposed to occupational noise and 60 non-exposed controls by applying TOAE and DPOAE to identify differences. The result was that military personnel not exposed to noise had higher TOAE recordings and DPOAE amplitudes compared to the noise-exposed group^14^.

Thus, it is important to study the hearing of military personnel that practice handgun shooting, and to inform these individuals about their own hearing, so that hearing preservation programs may be made for this population^15^,^16^.

The purpose of this study was to assess the knowledge about noise and the effects of impact noise on military personnel that practice handgun shooting, with the aim of implementing a hearing preservation program.

## MATERIALS AND METHODS

The institutional review board approved this study (no. 011/2009). Participants were given an explanation about the study and those that volunteered signed a free informed consent form before the study procedures were applied.

A case control study was carried out involving 115 military personnel, of which 65 belonged to the Special Operations Battalion (BOPE) and 50 were administrative staff from the Parana State Military Police (PMPR). The BOPE comprises about 100 military personnel, of which 65 agreed to participate.

The noise-exposed group consisted of BOPE military personnel that practiced handgun shooting regularly. The control group consisted of military administrative staff to have a homogenous as possible group with the exposed personnel for comparison purposes. Control group members could have been exposed occasionally to high-impact noise in their careers, but only those not involved in handgun shooting for more than 12 months and that had no hearing complaints were enrolled.

The mean age of participants in the exposed group was 32.2 years (23 to 44 years); the mean service time in this group was 9.1 years (1 to 25 years). The mean age of participants in the control group was 33 years (23 to 46 years); the mean service time in this group was 11.1 years (1 to 24 years). There were no significant differences in a comparison of the variables age (*p*=0.5165) and military service time (*p*=0.1136).

The first step was to assess the noise level of firearms that are used by the military personnel (pistols, revolvers, carbine, and rifles). Measurements were taken at the shooting outdoor training site with a sound pressure level measuring device (Brüel & Kjaer type 2230) on the compensation response circuit (C). Readings were taken close to the ear of shooters.

Military personnel were asked to answer a questionnaire about symptoms and care taken with hearing. Next, subjects underwent a physical examination and testing – inspection of the outer ear canal, air conduction pure tone audiometry at 250 to 8,000 Hz, and bone conduction pure tone audiometry (only if air auditory thresholds were higher than 25 dBHL, at 500 to 4,000 Hz HL), and TOAE and DPOAE. Subjects were acoustically at rest for auditory examination and testing.

Pure tone audiometry was done with an Interacoutics 229-B audiometer calibrated according to the ISO 8253-1 norm and TDH 39P earphones (air pathway) and B71 (bone pathway) in an acoustic booth, for frequencies ranging from 250 to 8,000 Hz bilaterally. Auditory thresholds up to 25 dBHL were considered normal. An Interacoutics Eclipse Platform device in an acoustic booth was used for TOAE and DPOAE testing. The pass/fail criterion was used in TOAE testing; up to 1,000 clicks at 75 dB were presented separately for each ear. The 3 dB algorithm method (reproducibility over 75% in at least three consecutive frequency bands with a signal-to-noise of at least 3 dB) was applied as a criterion for presence. The DP gram-extended method E, L1-L2 intensity of 10 dB with L1=65 dB and L2=55 dB, was applied in DPOAE testing (a f1/f2=1.22 ratio was used; the maximum test duration was 90 seconds). Testing comprised 1,000 to 8,000 Hz frequencies and their distortion products, which were considered present if the amplitude was above –10 dB and the signal-to-noise difference was greater or equal to 6 dB.

The following statistical procedures were applied in data analysis: Student's t test to compare questionnaire responses and audiometry, and the difference in proportions test to compare the results of TOAE and DPOAE. The significance level was 5% (0.05) in both cases.

All participants were included in the questionnaire analysis to check the general opinion and knowledge. Military personnel in both groups that had mixed or conductive hearing loss were excluded in the analysis of pure tone audiometry and OAE results.

## RESULTS

The firearm noise level in this study ranged from 119 to 133 dB (C). During training, at least 50 gunshots are made during 2 to 4 hours, consisting of a series of 5 to 10 shots separated by about 2-minute intervals.

The exposed group answered questions about the use of hearing protection devices during shooting practice. Nearly all subjects (92.3%) used hearing protection devices in shooting practice; about 70.7% used insertiontype ear protectors. Subjects reported that they had never been informed about the correct use of hearing protection devices (32.3%) or were given superficial instructions (38.6%). Other had been given such information only when entering the police force (12.3%); 16% said they had been well-informed. The available hearing protection devices were ear muffs (cup type) with no information about the brand, model, or sound reduction level. Each subject was able to choose and acquire other types of hearing protection devices.

**Graph 1:**
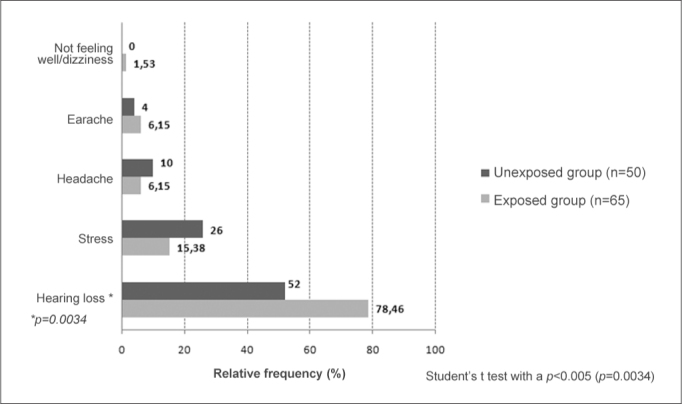
Comparison of awareness about the effects of noise in the exposed group (n=65) and not-exposed group (n=50).

Graph 1 shows the awareness of military personnel about the effects of noise on hearing. The groups differed significantly in their awareness that noise caused hearing loss; it was higher in the noise-exposed group.

The reported complaints of military personnel after shooting practice were tinnitus (23%), temporary hearing loss (7.6%), headache (3%), and irritability (3%).

**Graph 2:**
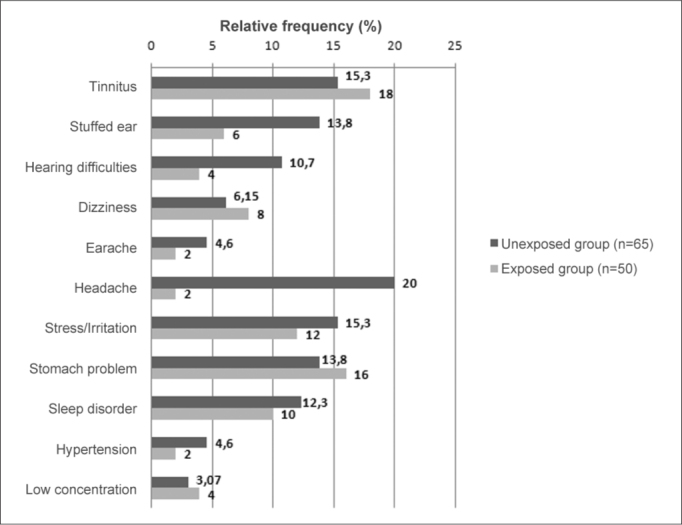
Auditory and extra-auditory complaints and symptoms in the exposed (n=65) and non-exposed (n=50) groups.

Graph 2 shows the auditory and extra-auditory symptoms and complaints reported in both groups.

In the exposed group, audiograms showed that nine military personnel had mixed or conductive auditory abnormalities; in the non-exposed group, two subjects had these findings, and were therefore excluded from the analysis.

Table 1 shows the audiometry results in the exposed group and the non-exposed group. The results were separated into normal auditory thresholds (up to 25 dB) and abnormal auditory threshold suggesting impact induced NIHL – a threshold with an acoustic notch at 3,000, 4,000 and/or 6,000 Hz were taken into account^2^. The differences in hearing profiles were statistically significant.

**Table 1:** Audiograms in the exposed group (GE) and the non-exposed group (GNE) to firearm noise (N=104).

Audiogram classification	Exposed group (n=56)	Non-Exposed (n=48)	*p*
Normal auditory thresholds	75% (42)	100%(48)	0.0001*
Suggestive of	25% (14)	0%(0)	0.0001*

* Student's t test; *p*< 0.05.

The rates of presence or absence of TOAE in the exposed group and the non-exposed group were calculated. Table 2 presents the results and shows that there was a statistically significant difference between groups.

**Table 2:** Results of TOAE in the exposed group (GE) and the non-exposed group (GNE) (N=104).

TOAE	GE (n=56)	GNE (n=48)	*p*
Present – bilateral	21.42% (12)	54.16% (26)	0.0008*
Absent – bilateral	58.92% (33)	35.41% (17)	0.0186*

* Student's t test; *p*< 0.05.

**Graph 3:**
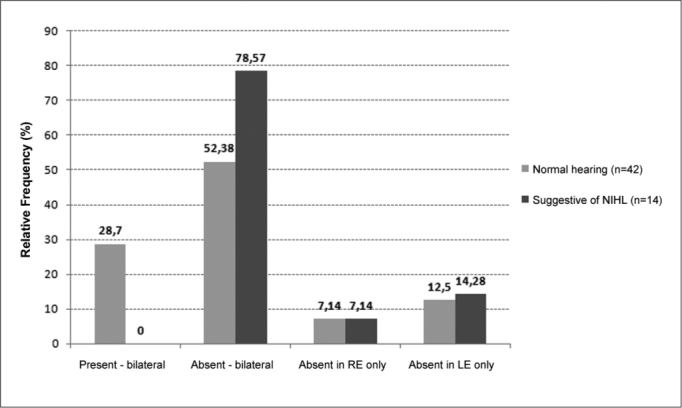
TOAE in the exposed group of subjects with normal audiograms and abnormal audiograms suggesting NIHL (N=56) (n=56).

Graph 3 shows TOAE test results between subjects in the noise-exposed groups according to whether their audiograms were normal or suggestive of NIHL. There was a higher rate of bilateral TOAE in normal hearing subjects. There were no differences between right and left ears.

**Graph 4:**
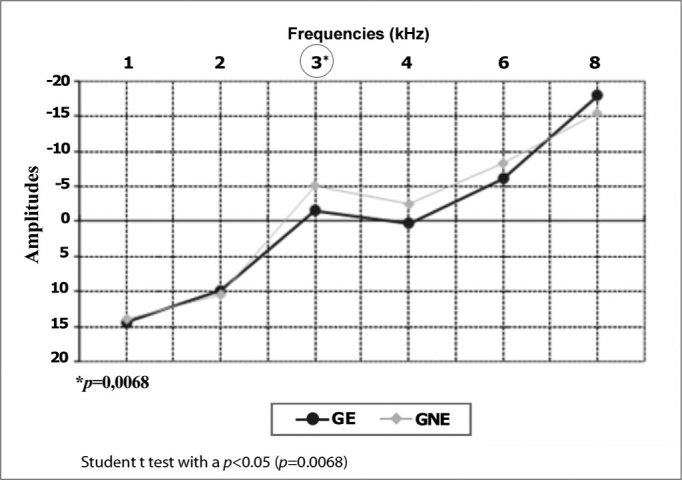
Comparison of DPOAE amplitude means in the right ears by frequency in the exposed (GE) and non-exposed group (GNE) (N=104).

**Graph 5:**
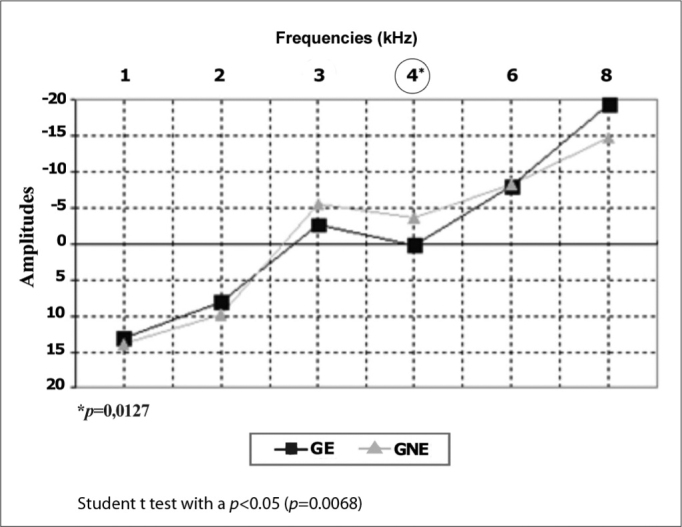
Comparison of DPOAE amplitude means in the left ears by frequency in the exposed (GE) and non-exposed group (GNE) (N=104).

DPOAE testing revealed a significant difference at 3 kHz in the right ear and at 4 and 8 kHz in the left ear. Graph 4, Graph 5 presents this amplitude difference among groups.

## DISCUSSION

Noise levels ranged from 119 to 133 dB (C). Other studies show similar results in firearm noise levels – 115.4 to 147.3 dB (C)^6^,^7^,^13^.

The military police force uses mostly the Pistol.40, which has a noise level of 124.9 dB(C). This level is higher than the limits in Annex II of the Regulatory Norm no. 15 (NR15)^17^, in which the tolerance limit for impact noise is 120 dB(C) with the sound pressure level set at compensation level “C”. The number of impacts relative to time in shooting training was higher than 50 impacts for 2 to 4 hours training (about 10 impacts in two minutes), which is also higher than the limit set in the NHO-01 (maximum exposure – 15 impacts per hour)^18^.

Most military personnel declared themselves aware of noise and 92.3% reported that they used hearing protection devices during shooting practice – mostly in-ear protectors (70.7%). This finding concurs with a Finnish study reporting that 86.1% of military personnel used hearing protection^19^, and a Brazilian study in Minas Gerais reporting that 94% of military personnel used hearing protection during shooting training^20^. However, a study in Sao Paulo found that only 35.4% of military personnel in the army use adequate hearing protection devices^21^.

Most subjects said that information given about hearing protection was superficial (38.4%); other reported never having received any guidance (32.3%). Although most military personnel reported using hearing protection, the effectiveness of these devices in preventing hearing loss is questionable. Hearing protection devices alone are often ineffective in avoiding harm to the hearing system; the result is progressive and permanent symptoms^20^,^21^. Ideally, each individual would have his own hearing protection device, as is the case of bullet-proof vests and helmets. In military circles, there is resistance against using hearing protection devices, especially in field work and combat; higher-ranking military personnel do not motivate their soldiers, as many believe that hearing protection reduces their safety^22^,^23^.

There was a significant difference (*p*=0.0034) between groups in awareness about the effects of exposure to noise impact; the exposed group was more aware of noise issues, as has been reported in the literature, especially because hearing protection devices are compulsory during shooting practice^24^.

The most frequent hearing complaints (at acoustic rest) in our sample were tinnitus and listening difficulties. According to the literature, these complaints are more frequent in military personnel that engage in shooting training^19^, ^20^, ^21^, ^22^,^25^.

Although symptoms were similar in the exposed and non-exposed groups to impact noise, hearing protection devices are effective. It is known that the sound attenuation level of a given hearing protection device depends on its physical characteristics and the person using that device; for instance, intermittent use drastically reduces its effectiveness. All subjects were aware of the effects of noise on hearing, and that hearing protection devices were the most frequently used method to avoid these effects, but many of them did not use these devices adequately; the reasons were difficulty to communicate, discomfort, and finding it impossible to use in certain settings^26^,^27^. The majority of subjects in the exposed group (92.6%) reported that they used hearing protection devices in all shooting practice sessions. Tinnitus was present in 23% of subjects soon after shooting practice, and in 15% when in acoustic rest.

The most frequent non-auditory symptoms in the noise-exposed group were headache (20% in the exposed group) and irritability (15.3% in the exposed group and 12% in the non-exposed group). There were no statistically significant differences between both groups in these or other symptoms, but there was a higher rate of these two symptoms in the exposed group. These findings concur with a survey of firemen in Sao Paulo, where irritability was found in 20.8% of cases, and headache was present in 14.5% of cases^10^. Another Brazilian study of workers exposed to noise showed that 16.9% had headaches and 11.3% reported irritation^28^.

The results of audiograms showed statistically significant differences between the exposed and non-exposed groups, and between military personnel with normal auditory thresholds and with notch-pattern sensorineural hearing loss at 3,000 and/or 4,000 and/or 6,000Hz suggesting NIHL. These findings also appear in other studies^9^, ^10^, ^11^,^29^.

Only 21.42% of noise-exposed military personnel had bilateral TOAE, compared with 54.16% in the non-exposed group. This difference was significant, indicating that frequent exposure to impact noise may injure outer hair cells. Further confirmation of this is seen in a comparison of noise-exposed subjects with audiograms suggesting NIHL and subjects with normal audiograms, in which TOAE are present at a higher rate compared to military personnel with NIHL. In this particular study, no subject with notch pattern sensorineural hearing loss in an audiogram had bilateral TOAE. Other studies have also shown that TOAE are absent at a higher rate in noise-exposed workers with normal pure tone audiometries, showing its efficacy in detecting cochlear hearing loss at an early stage^12^, ^13^, ^14^.

The amplitudes of DPOAE differed significantly at 3 kHz to the right and 4 kHz to the left. These frequencies are more sensitive to impact noise exposure^2^, ^3^, ^4^. A lower amplitude in the exposed group is a common finding in several studies of workers exposed to noise and military personnel involved in shooting practice, in which the amplitude of higher frequencies were lower in noise-exposed groups^12^, ^13^, ^14^, ^15^.

A proposal for an Auditory Preservation Program is to emphasize training for hearing protection device use, and to increase the awareness of military personnel about the importance of auditory health for his or her professional career and personal life.

## CONCLUSION

The health of military personnel exposed to firearms is at risk even when using hearing protection (92,3% of cases), as noise levels are high.

There are significant differences between the groups in pure tone thresholds (3.000, 4.000 and 6.000 Hz) and in OAE testing. Furthermore, the audiograms of 25% of military personnel exposed to firearm noise showed a notch-shaped sensorineural hearing loss.

The results of TOAE and DPOAE suggest that these tests are important and more efficient than audiometry alone for the early detection of cochlear injury due to firearm noise. Because these tests are fast, objective, and effective for detecting NIHL, they can be extra tests in occupational audiology for monitoring hearing in workers exposed to noise; these tests, therefore, are an additional tool for epidemiological surveillance in occupational health.

Further research is needed to extend this study, such as standardizing specific assessments for impact noise, periodic hearing monitoring of military personnel, and education for awareness about the use of hearing protection and protective measures for hearing.
